# Social hierarchies and social networks in humans

**DOI:** 10.1098/rstb.2020.0440

**Published:** 2022-02-28

**Authors:** Daniel Redhead, Eleanor A. Power

**Affiliations:** ^1^ Department of Human Behaviour, Ecology and Culture, Max Planck Institute for Evolutionary Anthropology, 04103 Leipzig, Germany; ^2^ Department of Methodology, London School of Economics and Political Science, London WC2A 2AE, UK

**Keywords:** social status, social hierarchy, social networks, social capital, social dynamics

## Abstract

Across species, social hierarchies are often governed by dominance relations. In humans, where there are multiple culturally valued axes of distinction, social hierarchies can take a variety of forms and need not rest on dominance relations. Consequently, humans navigate multiple domains of status, i.e. relative standing. Importantly, while these hierarchies may be constructed from dyadic interactions, they are often more fundamentally guided by subjective peer evaluations and group perceptions. Researchers have typically focused on the distinct elements that shape individuals’ relative standing, with some emphasizing individual-level attributes and others outlining emergent macro-level structural outcomes. Here, we synthesize work across the social sciences to suggest that the dynamic interplay between individual-level and meso-level properties of the social networks in which individuals are embedded are crucial for understanding the diverse processes of status differentiation across groups. More specifically, we observe that humans not only navigate multiple social hierarchies at any given time but also simultaneously operate within multiple, overlapping social networks. There are important dynamic feedbacks between social hierarchies and the characteristics of social networks, as the types of social relationships, their structural properties, and the relative position of individuals within them both influence and are influenced by status differentiation.

This article is part of the theme issue ‘The centennial of the pecking order: current state and future prospects for the study of dominance hierarchies’.

## 1. Introduction

Across many socially living species, individuals form social hierarchies. An individual’s position within such hierarchies reliably governs the extent of their social influence and access to group resources [1–3]. Many hierarchies observed across non-human animal communities are generated by agonistic interactions between individuals, which produce patterns of imbalance and create asymmetric dominance relationships within groups [4–6]. Success within such dominance hierarchies is determined by physiological differences, signals and heuristics that allow these properties of local interaction to construct relatively stable global hierarchies [7–9]. Analogous dynamics have been observed within human hierarchies, with certain individuals achieving and maintaining access to group resources through processes of intimidation, coercion, manipulation and aggression (see [10–14], for disciplinary reviews).

Social hierarchies are, however, highly multidimensional systems. An individual’s relative standing within a hierarchy is not always solely determined by their success across agonistic contests. Empirical evidence from a variety of non-human animals has highlighted that relative standing may be founded upon an individual’s competence [14] and leverage [15], and is also inherited [16]. Similar complexity has been observed among humans, where relative standing is commonly founded upon a composite of group members’ perceptions of their personal properties. A diversity of assets (e.g. material holdings; [17]) and individual qualities (e.g. skills and knowledge, prosociality; [18]) can help to establish a person’s relative standing. The perception of a person’s attributes and behaviour is then derived from the social information that people amass about one another—through direct or indirect interaction [19,20] and gossip [21]. Importantly, both the relevant qualities of individuals, and the perceptions of those qualities, are guided and constrained by broad cultural and socio-ecological factors.

In some contexts, human hierarchies are made particularly explicit (e.g. formal and institutionalised orders of precedence or seniority), while in others, they may be somewhat tacit, instead inferred from the actions and orientations of others. Humans, then, regularly form and navigate both formal and informal hierarchies. Within formal hierarchies (e.g. corporations, bureaucratic institutions, military groups), the positions of individuals are explicitly mandated, and the distribution of individual roles, responsibilities and social influence are officially sanctioned and widely known [11,22,23]. In informal hierarchies, the position of an individual cannot be directly observed. Rather, the differentiation of individuals is inferred through observations of their social interactions, assessments of their access to important group resources, and peer perceptions of an individual’s social influence, esteem and power [1,24].

Here, we define social hierarchies as fundamentally latent processes that describe social relationships between individuals and groups. By this definition, social hierarchies are inherently socio-relational phenomena; an individual cannot be high ranking without having a lower-ranking counterpart. Members of a group may confer status to those observably high in a given culturally valued attribute (or attributes) through a form of social exchange, deferring to the individual in the hope that it will help achieve personal goals [25–27]. These goals may be associated with desires for elevating one’s own relative standing and access to social, informational or material resources or for protecting one’s own interests and well-being [28]. Through processes of learning and decision-making, individuals tend to converge on their perceptions of others. This creates heuristics about the individual properties that delineate status [29,30]. Given this, the processes that determine who becomes high status—as well as the meaning and outcomes of what high or low relative standing entails—are highly context dependent and multi-modal, with individuals operating within different, co-existing hierarchies across their daily lives [31].

**Figure 1.**
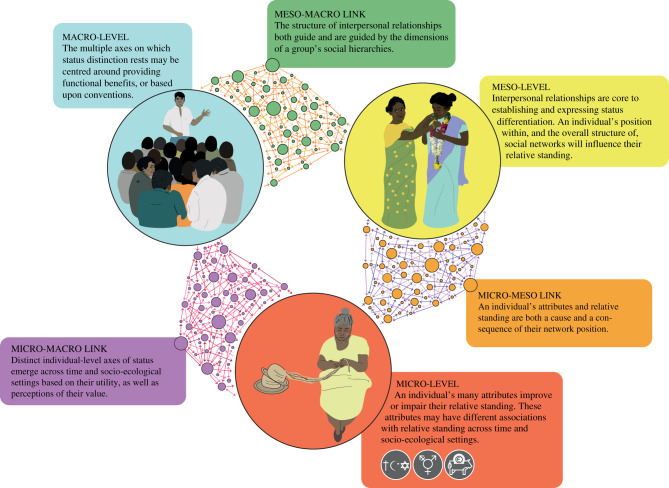
Schematic representation of the dynamic feedbacks operating across micro-, meso- and macro-levels that collectively shape social hierarchies in humans.

We first review the existing literature on social hierarchy among humans, which is typically focused on either the macro-level mechanisms or the micro-level factors (e.g. individual attributes) that govern status differentiation. We then outline an emerging body of work that has been largely overlooked in the evolutionary human sciences, which examines social hierarchy from a network perspective. In doing this, we outline how network properties can bolster or constrain the status of certain individuals and further highlight how such network characteristics can explain some of the variation in achievement of high social status observed across cultural and ecological contexts (see figure 1).

## 2. Macro-level processes and the emergence of social hierarchies

Recently, perspectives on why hierarchies emerge and persist across a diverse array of ecological and cultural contexts have begun to converge between the social and evolutionary sciences. Status differentiation, at its core, helps to facilitate coordination and collective action [32–35]. Problems of collective action and coordination abound across all socially living species and describe situations where individuals benefit from adjusting their own preferences and actions to align with the—potentially conflicting—actions and motivations of those around them [36,37]. Humans have a striking aptitude for participating in these collaborative actions that produce higher benefits to all involved parties than if they were to act alone [38,39]. For example, we coordinate irrigation across entire watersheds and follow abstract rules about which side of the road to drive on.

The emergence of effective coordination and collective action within groups is, however, challenging and can often be undermined by individuals acting in their own self interest. Such individuals—commonly termed ‘freeriders’ or ‘defectors’—may act selfishly and reap the benefits of a group’s collective efforts without providing their own contributions [40,41]. Theory and evidence suggest that groups tackle free riding and reduce the costs of coordination when certain individuals have disproportionate social influence (i.e. direct group efforts, and monitor the actions of others [42–44]) and when individuals who contribute to the collective effort are rewarded (be those rewards social or material [45,46]).

Through these processes, social status—both heightened social influence and greater access to resources—may well be attributed to individuals who have qualities, abilities or motivations that increase their capacity to provide benefits to other group members [13,19,28,47,48]. The qualities or abilities that are valued may vary across different socio-ecological settings, and so too, then, should the axes upon which status is apportioned. In contexts where skill is particularly fundamental to productive returns, status differentiation may rest on such distinctions, as with the importance given to skill and knowledge related to hunting in many hunter–gatherer societies [49]. Where coordination is particularly crucial for production or defence, charisma and oratorial skills may be foundational to status hierarchies [50,51]. In contrast, where returns to production are driven more by assets, rather than skill or time invested, as in many pastoralist and agriculturalist societies, delineations of wealth may be the crucial axis along which status is marked [52,53]. This wealth is often the substance of exchange and patron–client relationships, both marking status and also providing for the possibility of the redistribution of wealth (e.g. of cattle [54]). While the particular attributes or abilities that confer status are different across contexts, they could each be interpreted as providing functional benefits for all group members in the particular socio-ecological setting.

However, it may also be the case that status hierarchies rest not on obvious differences in ability, but rather on seemingly arbitrary distinctions. Individuals are better able to calibrate their behaviour when interactions are structured by rules, or behavioural regularities, that rest on easily distinguishable attributes [55]. These behavioural regularities are often termed *conventions* [56,57]. Just as language and driving on a specific side of the road are conventions that facilitate collective action and coordination, so too may social hierarchies rest on conventions. Rather than being adaptive responses to a particular aspect of the socio-ecology, then, the axes on which status differentiation is expressed may at some level be arbitrary, culturally learned conventions. Social hierarchy may have emerged as a near-universal form of social organisation because of its functional benefits, but that does not mean that the axes along which an individual’s relative standing are reckoned need always be functionally beneficial.

In many cases, an individual’s status is based not on attributes that directly impact their ability to provide benefits to others, but rather on markers of an individual’s social worth or on presumed correlates of perceived ability or competency. This creates a contrast between functionally beneficial status arising from actual ability and conventionally assigned status emerging from normative judgements of an individual’s worth. Consider, for example, artists, who may certainly be highly skilled, but whose work will be valued based on—and their status determined by—culturally specific assertions of worth.

This grounding in normative judgements highlights how status hierarchies (for humans in particular) may be based on subjective assessments that take into account the judgements of others. In line with this, social influence (i.e. our reliance on others’ assessments when forming our own) appears to play a key role in how closely status and ‘quality’ align. As people rely more on social information, the discrepancy between status and quality can grow [58,59]. And, once axes of status differentiation are established, they can readily become entrenched [60]. This may be true even if such axes cease to be meaningful in a particular socio-ecological context (e.g. [61]). Many evolutionary psychologists see such reliance on seemingly ‘defective’ cues or signals to an individual’s ability to provide benefits to others as cases of evolutionary ‘mismatch’ (e.g. [62]). However, it may be more plausible to interpret such conventions surrounding the conferral of status as emerging from processes of social learning (e.g. the formal theoretical literature examining the (cultural) evolution of inequality reviewed in [32]).

Regardless of whether an individual’s relative standing is derived from functionally beneficial attributes or heuristics based on conventions, status differentiation has seemingly emerged as an effective tool for overcoming problems of collective action and coordination. This is in part because, insofar as deference is given to those of relatively high status, those of high status may still be well positioned to influence others. It is important to note, however, that while many forms of social hierarchy observed among human groups do provide benefits to coordination and collective action, the extent of inequality within such hierarchies can be consequential (see [33]). In many cases, those with low relative standing will receive minimal benefits, and upward mobility across the hierarchy may be challenging. Alongside this, some human groups may, to a lesser extent, be organized within hierarchies that are determined through purely agonistic interactions [63]. Such hierarchies are centred on individual self-interest and the ability to inflict harm, providing only individual-level benefits for those able to reach high relative standing and have limited impact on a group's ability to coordinate or act collectively (although see [47], for evidence that such hierarchies may aid collective action in the context of inter-group conflict).

To further disentangle the processes that guide the emergence of these social hierarchies, we turn our attention to the empirical literature to establish what individual attributes are associated with increasing, or decreasing, an individual’s relative standing across societies.

## 3. The micro-level factors: individual attributes shape status differentiation

The desire to attain social status has been posited as a fundamental human motivation. Given this, theory is framed around individuals first aspiring to attain high social status and aiming to maintain their status once they occupy privileged positions within a hierarchy [64–66]. The reasoning behind these arguments is that having high relative standing grants a wealth of individual benefits, such as improved reproduction and survival (reviewed in [67,68]), and greater health status and subjective well-being (see [69], for a review). For the most part, the spotlight remains on trying to test hypotheses about the individual-level correlates of *high* status, with general neglect of the individual-level attributes, and macro-level constraints on such attributes, that precipitate *low* relative standing. For the remainder of this section, we overview the individual-level attributes that existing research has suggested to be important axes of status distinction.

### (a) Physiological cues

Empirical examinations of the antecedents of status often follow experimental designs where the physical characteristics of an individual—such as their facial symmetry, facial height-to-width radio and observable muscle mass—are manipulated. These manipulations aim to portray different social information about an individual’s qualities, such as their behavioural dispositions (e.g. their prosociality and cooperativeness [70–72]) or physical formidability (i.e. their ability to inflict harm or act with force [73–77]), which are proposed to impact relatively convergent impressions of their competence and leadership ability [78,79]. These studies provide some evidence as to the abstract physiological axes of, and preferences guiding, status distinction, and some of these features (e.g. physical strength and size) correlate with increased social status in observational settings (this has, however, only been investigated in samples of men [18,80]).

While these experimental examinations are often framed around relational narratives and aim to reduce complex status processes to manipulable quantities, their analytical framework generally treats status as an attribute of the individual. In doing so, a substantial amount of information about the properties of the individuals, and their relative standing, is abstracted to such a degree that the overall generalizability of the findings is highly constrained. Assessing individual attributes *in silo* may also place inordinate weight on characteristics that have limited importance in real-life settings. That is, many of these results may be robust when all else is held equal, but it is impossible for all else to be assumed constant, given that status is fundamentally a socio-relational process.

### (b) Age structure

Community elders are often accorded deference [81], enjoy positions of high relative standing [82] and occupy positions of leadership [83]. While age may not necessarily be a *causal* determinant of an individual’s relative standing, an individual’s age carries an abundance of important information about them. As knowledge and skills are built over the life course, age should reflect this accumulation. For example, ethnographic evidence has shown that age tracks individuals’ accumulated wealth among the Siuai [84] and culturally important knowledge among the Mekranoti [85].

This evidence suggests that status differentiation may have an age structure. Relative standing may be important only when comparing individuals of a similar age, who are vying for access to resources that are particularly meaningful at that stage in the lifespan. Thus, the axes in which status is differentiated may be unique to, and constrained by, the specific age class to which an individual belongs. For example, young adulthood may be when status competition is paramount, with individuals entering the market for sexual and marital partners and striving to forge a reputation that provides them solid grounding for success throughout their lifetime. Indeed, a correlation between high relative standing, marrying younger and having more marital partners has been shown among men in the Tsimane of lowland Bolivia [86].

### (c) Gender

Across the majority of human societies, men operate within public spheres and occupy more privileged positions of publicly visible status than women [87]. It is commonly assumed that women are unable to attain high relative standing in communities due to the sexual division of labour: with biological females acting as ‘carers’ and biological males as ‘competitors’ caused by differences in pay-offs and investment incentives [88]. Previous research has also emphasized that sexual dimorphism in physical strength and size, and sex-specific differences in how to best obtain and use resources for reproductive success (e.g. female-specific investment associated with reproduction) created universal patterns of male leadership and status elevation [87,89,90]. Importantly, however, in contexts where these constraints and associated trade-offs are effectively less severe, gender dynamics play out in very different ways (e.g. [91,92]).

While these biological constraints may have played some part in paving the way for status inequity between the sexes, the sexual division of labour is a product of complex co-evolutionary processes [93]. Women’s involvement in public status arenas is highly constrained by cultural beliefs and norms about gender [94]. Women may be as able and willing as men in any given status domain (e.g. they are able to provide functional benefits to others), but cultural conventions about expected gender-specific behaviours or attributes (e.g. women should express ‘communal’ behaviours, such as being sympathetic and gentle, while men should be agentic competitors [95]) and gender roles (e.g. women as the homemaker, and men as the responsible worker or authoritative leader [96]) may prejudice against high relative standing for women and female leadership [97,98]. Importantly, these cultural conventions may result in the same patterns of association between gender and status as would be predicted by sexual selection, despite very different underlying mechanisms. The observed patterns of gender differences in status may thus be the result of cultural evolutionary processes that favoured the use of easily observable—and potentially arbitrary—‘types’ (such as man/woman) as status heuristics [32], rather than being driven by any underlying biological differences between the genders.

### (d) Group identities

Other group identities—such as race or ethnicity, caste, sexual identity and social class—similarly facilitate or constrain status attainment [99,100]. Many human communities consist of different sub-groups, which are marked by seemingly arbitrary cultural traits (such as types of dress, speech [101]). These markers are thought to facilitate cooperation [102], by allowing individuals to easily assort with others who share their norms and values [103]. Conventions governing cooperation based on group identity can produce inequality between groups. Certain groups may obtain privileged positions based on these arbitrary distinctions, with or without any overt discrimination [104]. Which group gains a position of high standing may, for example, be determined by whether there are asymmetries between these groups, such as differences in their population size, strength and power, or wealth [32]. Through these processes, certain individuals may enjoy elevated status within their group or have constrained opportunities to better their social position [105–107].

### (e) Material wealth

Functional accounts of status differentiation often posit that status is conferred to those who are able to provide benefits to others [108]. Given this, individuals with a large amount of material wealth are commonly found to have high relative status. In an Inuit community in the Canadian Arctic, for example, those who can afford the expensive equipment necessary to harvest traditional foods (e.g. seal, caribou) can widely share their sizable yields, increasing their relative status within the community [109]. Substantial material wealth inequality has been observed across a variety of cultural and ecological settings [110], in part because wealth may be earned not only through an individual’s own labour but also through inheritance across generations [111].

Material wealth also allows individuals to acquire culturally valued goods, tastes or preferences [112]. These lend themselves to favourable perceptions of the individual’s relative standing within a community [113]. Those high in wealth are consequently perceived as being competent in culturally valued domains—regardless of whether they are actually competent—with others consequently conferring status to them [114]. These processes also lead to constraints to the relative status of individuals who lack wealth, as well as the cultural capital that accompanies it [115].

### (f) Personality and individual differences

Although attributes, such as material wealth, allow individuals to be *able* to confer benefits to others, this need not mean that they are *motivated* or *willing* to do so. Thus, an individual’s status may not be solely determined by whether they have amassed material wealth, but by how they use it. Indeed, research has suggested that individuals with more generous, agreeable and prosocial dispositions occupy positions of high relative standing [116,117]. Alongside this, studies have examined how certain personality traits are correlated with status [118–120]. For example, those high in self-esteem [121,122], self-monitoring [123,124] and extraversion [125] are often more likable, socially included, respected and conferred social status. However, fear, manipulation and coercive behaviours may also be positively correlated with status. These include narcissism [126] and different forms of dispositional aggression [127,128].

These individual-level correlates of social status are regularly conceptualized as broad, latent factors constructed from a composite of individual attributes [28,65,119]. There can be methodological and theoretical utility in doing so as the joint contribution of highly co-varying traits may be the target of analysis (e.g. composite’s of an individual’s ‘prestige’ or ‘dominance’ [19]). Various dimension reduction techniques are therefore often used, as high dimensional analysis may be intractable, as well as hard to interpret [129]. However, the correlations that many of these personality trait and individual differences have with status are often minimal across cultural and ecological settings. Many of these folk concepts have limited meaning, with their conceptual entities and measurable units taking myriad forms [130,131], and standard measurement instruments (e.g. Likert scales) used to measure these constructs do not transfer across contexts [132].

Many of the individual-level axes that we have discussed tend to be viewed as stable, time-invariant traits that have near-universal associations with status differentiation. This framing may be a consequence of the cross-sectional and experimental research designs used in many studies. However, the expression of many of these attributes, and their (bi-directional) associations with social status, likely vary over time [60]. This temporal variation may be not only due to underlying changes in attributes (e.g. a person’s wealth can change) but may also be due to changes in the composition of the population (e.g. a person is considered wealthy when they have more assets *compared to other group members*). The expression of any attribute or disposition (e.g. aggression) may be context specific (i.e. a person’s aggressiveness may change based on the set of people they are interacting with), meaning that individuals may be assessed differently by different observers [133].

## 4. The meso-level properties: social networks and status differentiation

Our attention so far, following the literature, has been on the individual attributes and qualities that play a role in determining a person’s position within social hierarchies. By conceptualizing social status as a socio-relational process, however, we highlight how these individual attributes (or personal resources) are socially contextualized and socially realized. The culturally desirable individual attributes, the emergence of status differentiation and the resources associated with high or low relative status are embedded within networks of social relationships and interactions [66,134,135]. Access to these social resources is often glossed as an individual’s *social capital* [66,112,136]. While popular, social capital as a concept remains nebulous, thanks to the multitude of definitions that it has received [137]. We approach social capital following Lin [66, p. 29], to mean ‘resources embedded in a social structure that are accessed and/or mobilized in purposive actions’. This includes tangible resources, such as borrowing a neighbour’s equipment, or more intangible ones, such as getting a mentor’s endorsement: either could be crucial in advancing a person’s relative position. Here, we review how different aspects of a person’s social connections and social position may also be associated with their relative status(es).

### (a) Social structure and social status

Social structure emerges from patterns of social interaction and social relationships within a population [136]. Social interactions are events in which sets of individuals (e.g. a dyad) engage in types of behaviours towards one another [5,138]. These behaviours may, for example, be moments of verbal or non-verbal communication or the exchange of goods. When individuals are involved in a series of interactions, they form perceptions of and sentiments towards their counterparts and create more generalized patterns of behaviour with one another [5,138,139]. That is, they form social relationships. Social relationships may, then, emerge informally through repeat engagements in particular types of interactions and enduring sentiments (e.g. friendships [140]), or be dictated by formal roles (e.g. in employment contexts, or kin relations [138]). Interactions and social relationships are rarely static or randomly patterned, and their temporal dynamics bring about observable global properties of a group. Here, we briefly outline the *direct associations* that interactions, social relationships and social structure have with status differentiation.

Among humans, and other animals, hierarchies can be built up from individual's perceptions not only of competitive interactions but also of cooperative exchanges of important resources [141]. These exchanges may be material (e.g. transfers of food, monetary loans) or informational (e.g. transmission of gossip or important cultural information). For example, in many hunter–gatherer societies, sharing the spoils of a hunt with others can be a key way to enhance a hunter’s relative standing [142,141]. Where material resources can be more readily accumulated, we see more pronounced patterns of status differentiation through generous acts, such as the *moka* exchanges seen in ‘big man’ societies in Melanesia [144]. Indeed, many societies have patron–client systems where a person’s standing is built and expressed through acts of largess [145].

As with social status, more subjective or cognitive representations of social relationships (i.e. who individuals believe to be their friends) are paramount for humans [139]. Importantly, social relationships are where social capital is embedded and are thus crucial for determining the structural properties of social hierarchies and the relative status of individuals [135]. Relationships of different strengths (e.g. acquaintance, best friend) and types (e.g. ally, kin) provide access to distinct material and informational resources [66]. For example, individuals may be more willing to loan money to a friend or relative and may not expect the loan to be repaid in full or within a strict time-frame, as they would with a stranger [146].

The social networks in which humans—and many socially-living species [147]—operate are often *multilayer* [148], with individuals navigating several social relationships at any point in time. The ‘layers’ within these networks can be complementary (e.g. friendship and advice-giving) with one relationship promoting the other, or competing, with one relationship precluding another (e.g. drug-sharing and employment [149]). In some cases, then, relationships can be a burden, creating obligations and constraining action. Therefore, individuals may be selective about the relationships they foster with others, as each distinct relationship is either directly or indirectly associated with certain important resources, and obligations, within their network(s). These inter-relations between network layers may thus be leveraged to attain and maintain status, with more *affective* relationships (e.g. friendship, kinship) providing a platform for, or constraining, *instrumental* connections (e.g. help finding a job), and vice versa [135].

### (b) Social capital and network position

#### (i) Direct connections

Alongside an individual’s personal properties, the structural position they occupy within their social networks may also shape status attainment. One of the clearest relational associates with status attainment is the number of relationships that an individual enjoys [150], referred to in network terms as *degree* centrality [151]. For example, in schools, higher status children and adolescents are often those who have the most friends [152], or are considered most popular among their peers [153,154]. Similarly, adults who have the most social support (e.g. food sharing and food production) partners are high status in several societies [98,155,156].

Having numerous partners can facilitate the acquisition and maintenance of high relative standing through the social capital that these relationships build [157]. This high relative standing has an important impact on many outcomes, as there is, for example, ample evidence of a strong positive relationship between social support and health [158–160]. Individuals can effectively call upon those who they have ties with—who are either motivated or obligated to contribute their resources—for social, political or material support [161]. By accessing the resources embedded within their personal network, well-connected individuals likely have greater, and more diverse, resources at their disposal [162]. Individuals with relatively fewer connections, in contrast, may find themselves more reliant on their own assets.

#### (ii) Network position

Partners may be important not only for the resources they hold but also for the positions they hold and the resources they can, in turn, access. Resources flow indirectly within networks as an individual may access and mobilize the resources of a mutual acquaintance (e.g. friends of a friend [163]). In a recent theoretical model of hierarchy formation, Kawakatsu *et al.* [164] highlight the importance of this process: interactions (or ‘endorsements’) produce stable hierarchies through social reinforcement, and interactions with better connected individuals provide greater contributions to an individual’s relative status than with those who are less connected. Empirical evidence of the inheritance of both social status and social connections in hyaenas suggests such processes may well be in operation in non-human animals as well [165]. In the context of social capital, this centrality related social reinforcement highlights the importance of access to, and mobilization across, many connections, as more privileged positions provide greater access to important resources. Empirically, this idea has been operationalized through several centrality metrics, such as *eigenvector* [166], *PageRank* [167] and *SpringRank* centrality [168].

Alongside this, individuals who are connected to multiple unconnected others may occupy privileged positions, as they serve as ‘brokers’ across the divide. Examples of this abound in formal hierarchies in organizational settings, where the hierarchy is fixed, and interactions are largely patterned by an individual’s official position. These examples often highlight the association between brokering information, and effective leadership and decision-making within companies [169,170]. ‘Brokerage’ has also been shown to influence relative standing in more dynamic networks, and informal social hierarchies. For instance, among the Orokaiva in Papua New Guinea, households positioned on the shortest paths between many other households within taro gift exchange networks (i.e. those with high *betweenness*) typically accumulate the most political and social support, as they have power to mediate resource flow and relationships between unconnected households [171]. By serving as such essential intermediaries, they are able to reap more of the rewards of whatever may flow through the network (whether informational or material [172,173]).

The idea of brokerage and bridging also extends to individuals who find themselves with connections between distinct and unconnected *groups*, bringing novel information and resources, and fostering advantageous connections and relationships between previously disconnected others [174–176]. These brokers have high relative status in contexts where interaction and relative standing are less formally mandated (e.g. [177]). For example, Matsigenka teachers in schools in and around Manu National Park in Peru often bridge communication, and facilitate the transmission of cultural norms, between Matsigenka and Mestizo communities. Given the unique resources (both material and informational) associated with this position, these teachers are highly respected and are elected as community representatives [178,179, pp. 113–115].

While, in certain contexts, bridging between unconnected groups may facilitate brokerage, such positions can also be fragile and met with suspicion, constraining an individual’s relative standing [176]. For example, fishers in Hawaii’s pelagic tuna fishery who bridged between ethnic groups, or smaller, structurally distinct groups, inspired lower trusted, and so were less economically productive [180]. The nature of relationships and setting is important to understanding the potential benefits—and costs—of brokerage. More generally, individuals who occupy peripheral positions within a network are likely to be constrained in their ability to improve their relative standing. For example, women often have limited access to well-positioned others and thus are less able to mobilize the valuable resources that are embedded more centrally within a network, which can crucially hinder their ability to build status [181,182].

#### (iii) Homophily and heterophily

Individuals not only associate with those of similar network positions but also selectively assort with others who possess similar, or the same, personal qualities to themselves (i.e. homophily [183,184]). In the context of status differentiation, individuals may preferentially associate with others who are of a similar status, or express similar levels of status-related individual attributes. This may be due to individual differences related to status differentiation producing distinct resources [185]. For example, there is ample evidence of status homogamy, i.e. homophily between marital partners, on the basis of educational attainment or earnings [186].

Patterns of heterophily (i.e. preference for making connections with *dissimilar* others) are also observed in social relationships in the context of status differentiation. There is reason to expect this, as individuals with low relative standing attach greater value to resources of high status counterparts, and thus compete to forge relationships and acquire access to such resources [187,188]. High status individuals, on the other hand, may prefer to connect with those lower in relative standing, given their greater bargaining power within such relationships [189], and are also likely competing with other high status individuals for these relationships to bolster perceptions of their relative value within their communities [156,161]. Heterophilous relationships may be beneficial to both parties, if status differentials align with different resources, roles or skills that are themselves complementary (e.g. a land owner and a gardener).

#### (iv) Network structure

Global attributes of a network may also influence status differentiation [190]. The extent to which others are aware of an individual’s actions and interactions should shape how consequential those are for altering their relative standing. In larger or sparser networks (i.e. low *density* networks where there are fewer connections between individuals), it may be harder for status-relevant information to circulate, leading to fragmentation in the assessments of an individual’s status. Similarly, networks with clear community structure (i.e. distinct clusters of individuals) may result in different communities variably observing and interpreting an individual’s actions and so arriving at different assessments of their relative standing. A community with a sparse network and weakly connected components may also find it hard to coordinate collective action, allowing for the few well-situated individuals to hold power and prevent any coalitions that might challenge them, effectively increasing inequality [191]. The extent to which resources and connections are concentrated around a few individuals (i.e. *network centralisation* and the shape of the degree distribution), and the linearity of the social hierarchy, may influence how readily an individual could improve their position. Through this, the structure and properties of a network can shape the global characteristics of a social hierarchy, and further constrain or bias individuals’ perceptions of status-related personal qualities of others within their network(s).

## 5. Feedback loops everywhere: the dynamics between the levels

Network-based frameworks for understanding human social hierarchy are distinct in their ability to couple the macro-level processes and the individual-level characteristics that shape an individual’s relative standing. These processes may be directly associated or may develop indirectly through meso-level properties of a network. For example, it is difficult in certain communities for women to attain public positions of high social status. This may be due not to cultural rules prohibiting female status attainment or to any sexually dimorphic trait that favours male physiology in producing public goods. Rather, gender differences in status attainment could materialize through indirect processes that constrain female relationships (e.g. ideas of modesty and honour that shape Bedouin women’s actions and interpersonal relationships [192]) and thus constrict a woman’s leverage within her social network(s). Particularly given the inherently relational nature of status, it is important to consider how feedbacks across scales may further shape status hierarchies [109].

### (a) Success breeds success

Moreso than in other species, humans’ assessment of status is shaped by perception and the (collective) social assessment of performance and value. This social influence can readily lead to a positive feedback loop where success breeds success [193], often referred to as the *Matthew Effect* [194,195]. This cumulative advantage can lead not only to the entrenchment of status differentials but also to status dispersion, as well-positioned individuals compound the benefits of their station [59,187,196,197], all of which can further privilege already advantaged groups [198]. Similar feedbacks may be in operation for individuals who find themselves in advantaged network positions. For example, individuals who broker connections between disconnected groups and thus harbour useful resources may become central to their primary network(s) over time. Importantly, these advantages may help individuals acquire the attributes that facilitate status advancement, as when social capital builds human capital [136]. The corollary here may also be true: those who are relatively marginalized or of lower standing may face greater hurdles to achieving status, regardless of their attributes. They may face a ‘reputational poverty trap’ [199] where they are less able to reap the reputational and status benefits of their actions.

### (b) Homophily revisited: network selection and network influence

As aforementioned, theory suggests that individuals tend to assort with those who are similar to themselves. The effects of this assortment may compound over time or generations to increase inequality in status [197,200]. While status-based homophily, also referred to as *network selection*, may certainly shape the observed patterns of similarity between connected individuals, a distinct mechanism, *network influence* (or ‘contagion’), can also cause this observed similarity [201]. That is, individuals may not preferentially form relationships to similar others but, over time, may become more similar to their partners because of their relationship [202,203]. This is especially important to consider when assessing the causal mechanisms creating similarity in the context of social capital (reviewed in [204]), where individuals have the resources of their connections at their disposal. This process was examined in a longitudinal study among the Tsimane [156], where von Rueden *et al*. highlighted that individuals may be motivated to form certain relationships (e.g. food sharing, alliance formation) with status-dissimilar others through mutual aspiration to increase (or, for those high in status, maintain) their status. Through the social capital that such ties create—especially for the lower-status counterpart—the statuses of these individuals become increasingly similar over time. Consequently, observed similarity between individuals could be as a product of either network selection or network influence, or both.

Clearly, an individual’s qualities, assets and network position will shape their ability to attain high social status. Above, we reviewed the substantial empirical evidence of strong positive associations between such elements and achieved status. But we also suggested that, in humans in particular, there is ample room for status hierarchies to be shaped by things *other* than the attributes nominally determining its structure, most notably, the social relationships that link individuals together. Not only can those relationships directly benefit individuals, as we outlined earlier, but so too can people’s perceptions of such relationships and statuses [205]. The knowledge that others choose to associate with a person or accord them status can help make an individual’s status more widely visible to the group and further increase the likelihood of these other group members conferring status to that individual [156]. This use of social information and the referential feedbacks it can produce can therefore also help to sustain social hierarchies, whether based on conventions or on functionally beneficial attributes. It is therefore crucial to consider the dynamic feedbacks—operating across scales—that produce observed status hierarchies.

## 6. Conclusion and future directions

The aim of this review has been to frame status differentiation as a socio-relational process and outline the extant literature that draws attention to the dynamics of social hierarchy. In following the literature, our review has highlighted the macro-level processes and micro-level factors that shape status differentiation across human societies. We have argued that while social hierarchy may provide functional benefits for coordination and collective action, the particular axes along which status is differentiated need not be functionally beneficial and can instead be culturally learned conventions. We further extend the literature by integrating theory and evidence of the meso-level properties that are key to linking these micro- and macro-level processes (see figure 1). This has highlighted the utility of taking a network-based approach to understanding and investigating human social hierarchies. In doing this, we hope to inspire future research that embraces that complexity associated with the dynamics of human social hierarchy and considers status differentiation as an ongoing process.

We see many fruitful avenues for further work building on this perspective. First, detailed comparative work should be conducted to document and assess the meaning of social hierarchies across cultural and ecological settings. Comparison could also be fruitful across different species: many of the complexities explored here are likely not unique to humans but may also occur in other social living species. This comparative work may further unpack the factors that have brought about observed similarities and differences in the axes of status differentiation across human (and non-human) communities.

Second, future research should explore how status is enacted in practice. Ethnographic (and ethological) research can provide rich detail on how individuals navigate the various status hierarchies that they are embedded in, and how those are entangled with other aspects of sociality. How does status differentiation articulate with the closely related concept of leadership (see the recent discussion in [44,206,207])? And how might the association between status and leadership vary across contexts? These questions will require observational work, which will be essential, but inevitably challenging, given the many complexities and feedbacks we have outlined here.

Longitudinal research will be particularly crucial for future research to examine the dynamics feedbacks that we have outlined. Having longitudinal data allows researchers to examine how processes of status differentiation operate across different timescales. Through this, such research is better able to determine how functional and conventional forms of status differentiation may emerge within groups. Alongside this, longitudinal research can further establish the age structure of social hierarchies and characterize status dynamics across an individual’s life course.

The practicalities of exploring these research avenues are daunting, but have recently become more feasible. Advanced tools for data collection (e.g. [208]) and network inference are continually being developed and refined. Generative network models, such as exponential random graph models [209], stochastic actor-oriented models [210], and latent network frameworks [211] hold particular promise, as they allow for the simultaneous consideration of multiple mechanisms operating across scales. New tools that allow for yet more complex network structure (e.g. multilayer network data) are also being rapidly developed, holding new promise for the field (e.g. [212]). Useful guides introducing these new methods and their utility are an important resource for researchers interested in taking up this call (e.g. [213–217]). By incorporating these methodological advances, future research will provide important advances to our understanding of how status is attained and maintained, and which factors shape the diversity of social hierarchies observed among human and non-human groups.

## Data accessibility

This article has no additional data.
